# Association of Early Physical Therapy With Long-term Opioid Use Among Opioid-Naive Patients With Musculoskeletal Pain

**DOI:** 10.1001/jamanetworkopen.2018.5909

**Published:** 2018-12-14

**Authors:** Eric Sun, Jasmin Moshfegh, Chris A. Rishel, Chad E. Cook, Adam P. Goode, Steven Z. George

**Affiliations:** 1 Department of Health Research and Policy, Stanford University School of Medicine, Stanford University, Stanford, California; 2 Department of Anesthesiology, Pain, and Perioperative Medicine, Stanford University School of Medicine, Stanford, California; 3 Center for Health Policy, Stanford University School of Medicine, Stanford, California; 4 Center for Primary Care and Outcomes Research, Stanford University School of Medicine, Stanford, California; 5 Duke Clinical Research Institute, Department of Orthopaedic Surgery, Duke University School of Medicine, Durham, North Carolina

## Abstract

**Importance:**

Nonpharmacologic methods of reducing the risk of new chronic opioid use among patients with musculoskeletal pain are important given the burden of the opioid epidemic in the United States.

**Objective:**

To determine the association between early physical therapy and subsequent opioid use in patients with new musculoskeletal pain diagnosis.

**Design, Setting, and Participants:**

This cross-sectional analysis of health care insurance claims data between January 1, 2007, and December 31, 2015, included privately insured patients who presented with musculoskeletal pain to an outpatient physician office or an emergency department at various US facilities from January 1, 2008, to December 31, 2014. The sample comprised 88 985 opioid-naive patients aged 18 to 64 years with a new diagnosis of musculoskeletal shoulder, neck, knee, or low back pain. The data set (obtained from the IBM MarketScan Commercial database) included person-level *International Classification of Diseases, Ninth Revision* or *Tenth Revision* diagnosis codes, *Current Procedural Terminology* codes, and date of service as well as pharmaceutical information (National Drug Code, generic name, dose, and number of days supplied). Early physical therapy was defined as at least 1 session received within 90 days of the index date, the earliest date a relevant diagnosis was provided. Data analysis was conducted from March 1, 2018, to May 18, 2018.

**Main Outcomes and Measures:**

Opioid use between 91 and 365 days after the index date.

**Results:**

Of the 88 985 patients included, 51 351 (57.7%) were male and 37 634 (42.3%) were female with a mean (SD) age of 46 (11.0) years. Among these patients, 26 096 (29.3%) received early physical therapy. After adjusting for potential confounders, early physical therapy was associated with a statistically significant reduction in the incidence of any opioid use between 91 and 365 days after the index date for patients with shoulder pain (odds ratio [OR], 0.85; 95% CI, 0.77-0.95; *P* = .003), neck pain (OR, 0.92; 95% CI, 0.85-0.99; *P* = .03), knee pain (OR, 0.84; 95% CI, 0.77-0.91; *P* < .001), and low back pain (OR, 0.93; 95% CI, 0.88-0.98; *P* = .004). For patients who did use opioids, early physical therapy was associated with an approximately 10% statistically significant reduction in the amount of opioid use, measured in oral morphine milligram equivalents, for shoulder pain (−9.7%; 95% CI, −18.5% to −0.8%; *P* = .03), knee pain (−10.3%; 95% CI, −17.8% to −2.7%; *P* = .007), and low back pain (−5.1%; 95% CI, −10.2% to 0.0%; *P* = .046), but not for neck pain (−3.8%; 95% CI, −10.8% to 3.3%; *P* = .30).

**Conclusions and Relevance:**

Early physical therapy appears to be associated with subsequent reductions in longer-term opioid use and lower-intensity opioid use for all of the musculoskeletal pain regions examined.

## Key Points

**Question:**

Is early physical therapy associated with long-term opioid use by patients with musculoskeletal pain?

**Findings:**

In this cross-sectional analysis of 88 985 patients with shoulder, neck, knee, or low back pain, early physical therapy was associated with an approximately 10% statistically significant reduction in subsequent opioid use.

**Meaning:**

By serving as an alternative or adjunct to short-term opioid use for patients with musculoskeletal pain, early physical therapy may play a role in reducing the risk of long-term opioid use.

## Teaser

This cross-sectional study examines the association between early physical therapy and subsequent opioid use among opioid-naive adults with a new diagnosis of musculoskeletal shoulder, neck, knee, or low back pain.

## Introduction

Musculoskeletal pain poses a tremendous health burden in the United States, with estimates suggesting that it affects nearly 1 of 2 adults and incurs an annual cost of $874 billion.^1^ A particularly concerning aspect of musculoskeletal pain is the potential for patients to transition to chronic opioid use, with prescription opioids playing a role in more than a third of drug overdose deaths.^2^ Both the US Centers for Disease Control and Prevention^3^ and the American College of Physicians^4^ now advocate for nonpharmacologic pain management as the frontline option for the management of chronic pain, including musculoskeletal pain.

Early use of physical therapy holds the promise of reducing opioid use among patients with musculoskeletal pain. In particular, by serving as an alternative or adjunct to short-term opioid use for episodes of acute musculoskeletal pain, early use of physical therapy may reduce total opioid exposure. In addition, by concurrently addressing physical impairments, physical therapy may present functional gains that decrease long-term opioid use. In the case of low back pain (LBP), previous work has found an equivocal association between the use of early physical therapy and patient-reported outcomes, health care costs, and opioid use.^5,6,7,8,9,10,11^ Moreover, the implication of early physical therapy for other highly prevalent musculoskeletal pain conditions (eg, neck, knee, and shoulder pain) is largely unknown.

Given the recent convergence in national priorities, a broader investigation of nonpharmacologic pain management for LBP and other prevalent types of musculoskeletal pain, such as neck and knee pain, is warranted.^3,4^ In particular, an important issue given recent guideline recommendations^3,4^ is whether early physical therapy is associated with reduced long-term opioid use for patients with musculoskeletal conditions.^12^ Using a large database of health care claims for privately insured patients, we examined whether early physical therapy was associated with reductions in long-term opioid use. Our analysis focused on patients with low back, neck, knee, or shoulder pain that was severe enough to require 2 visits to a physician within 30 days and at least 1 opioid prescription during the 90 days after the initial diagnosis.

## Methods

### Data

The study data consisted of insurance claims between January 1, 2007, and December 31, 2015, obtained from the IBM MarketScan Commercial database. The MarketScan database provides person-level data on health care use and expenditures for people enrolled in private insurance plans through a participating employer, health plan, or government organization. For inpatient and outpatient encounters, the database provides information such as *International Classification of Diseases, Ninth Revision* or *Tenth Revision,* (*ICD-9* or *ICD-10*) diagnosis codes, *Current Procedural Terminology* (*CPT*) codes, and date of service. For pharmacy claims, the database provides details such as National Drug Code, generic name, dose, and the number of days supplied. These data are frequently used in analyses of health care use and spending.^13,14,15,16^ This study received approval from the Institutional Review Board at Stanford University, which also issued a waiver of consent. Patient consent was waived because the data used were deidentified. This study followed the Strengthening the Reporting of Observational Studies in Epidemiology (STROBE) reporting guidelines for cross-sectional studies. Data analysis was conducted from March 1, 2018, to May 18, 2018.

### Sample

We constructed a sample of all adult patients (aged 18-64 years) who presented to a physician office (outpatient visit) or an emergency department at various US facilities with musculoskeletal neck, knee, shoulder, or low back pain between January 1, 2008, and December 31, 2014. We identified these visits by identifying claims with (1) a *CPT* code for an outpatient visit and/or a *CPT* code for an emergency department visit (eAppendix and eTable 1 in the Supplement) and (2) a primary *ICD-9* diagnosis code for musculoskeletal neck, knee, shoulder, or low back pain (eAppendix and eTable 2 in the Supplement).

For each patient, we defined the index date as the earliest date a relevant diagnosis was received and the study period as the time from the year before the index date to the year after the index date. We restricted our analysis to patients (1) who were continuously enrolled in a preferred provider organization or traditional indemnity plan with prescription drug coverage for the entire study period and (2) who were opioid naive, defined as having filled no prescription for an opioid in the 12 months prior to the index date. In addition, because musculoskeletal pain is a common diagnosis that can represent varying levels of severity, we restricted our analysis a priori to patients whose pain was severe enough to result in (1) a second outpatient visit or emergency department visit with a primary diagnosis of musculoskeletal pain within 30 days after the index date and (2) an opioid prescription within 90 days after the index date.

Using these criteria, we created an initial sample of 177 050 patients. We then applied the following exclusion criteria: younger than 18 years or older than 64 years as of the index date (n = 5131); an inpatient admission with a primary *ICD-9* diagnosis code for musculoskeletal pain or an inpatient admission with any *ICD-9* diagnosis code corresponding to an accident or a trauma during the study period (all diagnosis codes starting with an E), to exclude potential trauma patients (n = 13 259); a cancer diagnosis during the study period (n = 5990); and musculoskeletal pain in more than 1 region during the study period (eg, neck and shoulder pain; n = 40 523). We imposed this latter restriction because individuals with pain in multiple sites may represent a distinct patient population and a different set of disease processes compared with individuals with single-site pain. We excluded individuals with LBP who had another diagnosis that could be mistaken for musculoskeletal pain (n = 19 799) (eg, osteomyelitis; eAppendix and eTable 3 in the Supplement) as well as individuals with missing data on opioid use (n = 2387). To avoid extreme outlier and potential data error factors, we excluded the top 1% of opioid users, as measured by oral morphine milligram equivalents (MMEs) used between 0 and 90 days as well as between 91 and 365 days after the index dates (n = 976); these cutoffs amounted to 4455 MMEs between 0 and 90 days and 18 100 MMEs between 91 and 365 days after the index date. The final sample consisted of 88 985 patients (eAppendix and eFigure in the Supplement).

### Exposure

The exposure of interest was whether the patient received early physical therapy, defined as at least 1 physical therapy session within 90 days of the index date. Early physical therapy was measured by identifying claims with a relevant *CPT* code (97001, 97002, 97110, 97140, 97124, and 97150) and an accompanying *ICD-9* diagnosis code that corresponded to the patient’s pain site.

### Outcomes

The primary outcome consisted of opioid use, measured as an opioid prescription or measured in oral MMEs between 91 and 365 days after the index date. We isolated prescriptions for an opioid (hydrocodone bitartrate, hydromorphone hydrochloride [oral], methadone hydrochloride, morphine sulfate [oral], oxymorphone hydrochloride, and oxycodone hydrochloride) during the aforementioned study period, using pharmacy claims data. We converted each prescription to MMEs according to the reported drug dose and quantity supplied.^17^

### Additional Variables

We included additional variables to adjust for potential confounding. First, we adjusted for age and sex, which are directly provided in the claims data. Second, using claims submitted during the year prior to the index date (1-365 days before the index date), we used previously described methods^18^ to measure the presence of the medical comorbidities (eg, diabetes, hypertension) that compose the Elixhauser index, an index that is frequently used for risk adjustment.^18,19^ Third, we measured opioid use (MMEs) during the first 90 days after the index date.

### Statistical Analysis

Descriptive statistics in the form of means and 95% CIs were used to describe the distribution of demographics and comorbidities across each group. To assess the differences in characteristics between patients who received early physical therapy and those who did not, we used an unpaired, 2-tailed *t* test for continuous variables (eg, age) and a χ^2^ test for discrete variables (eg, comorbidities). We used a 2-sided *P* ≤ .05 to assess the statistical significance of differences between the 2 groups. Because of the large sample, even trivially small differences may be statistically significant; thus, we used Hedge g to estimate the magnitude of the standardized difference between the 2 groups. Hedge g is the difference between the means of 2 groups divided by the population SD,^20^ with values of less than 0.2 typically representing small differences between the 2 groups, between 0.2 and 0.5 representing moderate differences, and greater than 0.5 representing large differences.^21^

Next, we estimated the association between early physical therapy and the primary outcome (opioid use between 91 and 365 days after the index date) by using multivariable regression models. Most patients (76%) in the sample did not use any opioid at all during this period. When there are many observations for which the dependent variable (ie, opioid use between 91 and 365 days after the index date) is 0, a simple regression analysis tends to be downward biased (ie, the estimated effect is lower in magnitude than the true effect).^22^ To address this issue, we performed a 2-step analysis. The first analysis consisted of a multivariable logistic regression in which the dependent variable was whether the patient used any opioid at all and the independent variable of interest was whether the patient received early physical therapy. Therefore, the first analysis estimated the association between early physical therapy and the subsequent use of any opioid at all.

The second analysis consisted of a multivariable linear regression in which the dependent variable was the actual MMEs used between 91 and 365 days and the independent variable of interest was whether the patient received early physical therapy. This second analysis was restricted to patients who used opioids between 91 and 365 days after the index date (ie, patients for whom the MME was greater than 0). The analysis estimated the association between early physical therapy and the intensity of opioid use among patients who used opioids. This 2-step approach has been used in other studies to obtain nonbiased estimates when a large proportion of observations assumed a value of 0.^23,24,25^

For both the logistic and linear regressions, we included adjustments for potential confounders, including age, sex, year of diagnosis, patient comorbidities (shown in Table 1), and opioid use (in MMEs) during the first 90 days after the index date. These statistical analyses incorporated robust SEs and were performed using Stata MP, version 14.0 (StataCorp LLC).

**Table 1.** Difference in Early Physical Therapy Use Across Pain Sites

Pain Site	Early Physical Therapy During First 90 d After Diagnosis, No. (%)	Median (IQR)	
		Days Until First Physical Therapy Session	Early Physical Therapy Sessions During First 90 d After Diagnosis
Shoulder (n = 8579)	2954 (34)	40 (19-60)	6 (3-10)
Neck (n = 17 440)	6505 (37)	13 (4-26)	8 (3-13)
Knee (n = 22 083)	4401 (20)	34 (17-54)	5 (2-9)
Low back (n = 40 883)	12 236 (30)	15 (6-29)	5 (2-9)

Abbreviation: IQR, interquartile range.

### Sensitivity Analyses

We examined the robustness of the study results as applied to alternative specifications. First, to characterize the specific period when physical therapy may be advantageous, we performed a sensitivity analysis in which we divided patients who received early physical therapy into 2 groups: those who received physical therapy in the first 30 days after the index date, and those who received physical therapy between 31 and 90 days after the index date. We then estimated the association between these 2 groups and opioid use between 91 and 365 days after the index date, using the previously described statistical approach. Second, we performed an analysis in which we raised the threshold for early physical therapy to require 2 or more physical therapy visits within the first 90 days after the index date. Third, we conducted a sensitivity analysis in which we considered an alternative outcome: chronic opioid use, defined as having filled 10 or more prescriptions or filled the 120 or more days’ supply between 91 and 365 days after the index date.^26^

## Results

The final sample of 88 985 patients consisted of 51 351 (57.7%) men and 37 634 (42.3%) women with a mean (SD) age of 46 (11.0) years. Of this sample, 26 096 patients (29.3%) received early physical therapy, and the use of early physical therapy increased from 3318 of 12 262 patients (27.1%) in 2008 to 2706 of 8705 patients (31.1%) in 2014. The numbers of patients for each pain site are shown in Table 1. The median (interquartile range [IQR]) days until the first physical therapy session ranged from 13 (4-26) days for neck pain to 40 (19-60) days for shoulder pain, and the median (IQR) number of physical therapy session ranged from 5 (2-9) sessions for knee pain and LBP to 8 (3-13) sessions for neck pain.

Patients who received early physical therapy, compared with patients who did not receive early physical therapy, were slightly younger (mean [SD] age 45.7 [10.8] years vs 46.0 [11.1] years; *P* < .001; Table 2) and less likely to be male (14 702 [56.3%] vs 36 649 [58.3%]; *P* < .001). For 15 of the 28 comorbidities that we examined, no statistically significant differences were found across the groups. For the remaining 13 comorbidities, 1 comorbidity (depression) was more prevalent among patients receiving early physical therapy, whereas the other 12 comorbidities (eg, obesity and alcohol abuse) were more prevalent among the group of patients who did not receive early physical therapy. However, the magnitude of all of these differences was small, with a Hedge g of 0.05 or less for all characteristics.

**Table 2.** Sample Summary Statistics

Variable	No. (%) [95% CI, %]		*P* Value	Hedge g^a^
	With Early Physical Therapy (n = 62 889)	Without Early Physical Therapy (n = 26 096)		
Age, mean (SD), y	46.0 (11.1)	45.7 (10.8)	<.001	0.03
Male sex	36 649 (58.3) [57.9-58.7]	14 702 (56.3) [55.7-56.9]	<.001	0.04
Congestive heart failure	368 (0.6) [0.5-0.6]	116 (0.4) [0.4-0.5]	.009	0.02
Arrhythmia	1440 (2.3) [2.2-2.4]	548 (2.1) [1.9-2.3]	.08	0.01
Valvular disease	883 (1.4) [1.3-1.5]	352 (1.4) [1.2-1.5]	.52	<0.01
Pulmonary circulation disorders	142 (0.2) [0.2-0.3]	50 (0.2) [0.1-0.2]	.32	0.01
Peripheral vascular disease	412 (0.7) [0.6-0.7]	198 (0.8) [0.7-0.9]	.09	0.01
Hypertension				
Uncomplicated	13 360 (21.2) [20.9-21.6]	5022 (19.2) [18.8-19.7]	<.001	0.05
Complicated	571 (0.9) [0.8-1.0]	315 (0.8) [0.7-0.9]	.13	0.01
Paralysis	45 (0.07) [0.05-0.09]	16 (0.06) [0.03-0.09]	.60	<0.01
Other neurologic disorders	519 (0.8) [0.8-0.9]	225 (0.9) [0.8-1.0]	.86	<0.01
Chronic obstructive pulmonary disease	3758 (6.0) [5.8-6.2]	1374 (5.3) [5.0-5.5]	<.001	0.03
Diabetes				
Uncomplicated	5500 (8.7) [8.5-9.0]	2014 (7.8) [7.5-8.1]	<.001	0.03
Complicated	869 (1.4) [1.3-1.5]	315 (1.2) [1.1-1.3]	.04	0.02
Hypothyroidism	3805 (6.1) [5.9-6.2]	1661 (6.4) [6.1-6.7]	.08	0.01
Renal failure	328 (0.5) [0.5-0.6]	101 (0.4) [0.3-0.5]	.008	0.02
Liver disease	700 (1.1) [1.0-1.2]	303 (1.2) [1.0-1.3]	.54	<0.01
Peptic ulcer disease	97 (0.2) [0.1-0.2]	55 (0.2) [0.2-0.3]	.06	0.01
AIDS/HIV	64 (0.10) [0.08-0.13]	38 (0.2) [0.1-0.2]	.08	0.01
Rheumatoid arthritis/collagen vascular disease	764 (1.2) [1.1-1.3]	325 (1.3) [1.1-1.4]	.71	<0.01
Coagulopathy	220 (0.4) [0.3-0.4]	86 (0.3) [0.3-0.4]	.64	<0.01
Obesity	1825 (2.9) [2.8-3.0]	671 (2.6) [2.4-2.8]	.007	0.02
Weight loss	255 (0.4) [0.4-0.5]	98 (0.4) [0.3-0.5]	.52	<0.01
Fluid and electrolyte disorders	767 (1.2) [1.1-1.3]	295 (1.1) [1.0-1.3]	.26	<0.01
Anemia				
Blood loss	89 (0.1) [0.1-0.2]	37 (0.1) [0.1-0.2]	.99	<0.01
Deficiency	667 (1.1) [1.0-1.1]	302 (1.2) [1.0-1.3]	.21	<0.01
Abuse				
Alcohol	367 (0.6) [0.5-0.6]	116 (0.4) [0.4-0.5]	.01	0.02
Drug	201 (0.3) [0.3-0.4]	56 (0.2) [0.2-0.3]	.008	0.02
Psychoses	138 (0.2) [0.2-0.3]	51 (0.2) [0.1-0.3]	.005	<0.01
Depression	4230 (6.7) [6.5-6.9]	1893 (7.3) [6.9-7.6]	.005	0.02

^a^ Hedge g is a measure of the magnitude of any differences between the 2 groups, with values less than 0.2 typically representing small differences.

The unadjusted odds ratio (OR) of receiving 1 or more opioid prescription between 91 and 365 days after the index date for patients with early physical therapy was 0.91 (95% CI, 0.82-1.00; *P* = .06) for shoulder pain, 0.94 (95% CI, 0.88-1.02; *P* = .13) for neck pain, 0.87 (95% CI, 0.80-0.94; *P* < .001) for knee pain, and 0.99 (95% CI, 0.95-1.04; *P* = .78) for LBP (Table 3). After adjusting for potential confounders (eg, medical comorbidities), we found that early physical therapy was associated with a statistically significant reduction in the incidence of any opioid use between 91 and 365 days for patients with shoulder pain (OR, 0.85; 95% CI, 0.77-0.95; *P* = .003), neck pain (OR, 0.92; 95% CI, 0.85-0.99; *P* = .03), knee pain (OR, 0.84; 95% CI, 0.77-0.91; *P* < .001), and LBP (OR, 0.93; 95% CI, 0.88-0.98; *P* = .004).

**Table 3.** Subsequent Opioid Use After Early Physical Therapy

Pain Site	Any Opioid Use Between 91 and 365 d After Index Date					Change in Opioid Use Between 91 and 365 d After Index Date				
	Unadjusted OR (95% CI)	*P* Value	Adjusted OR (95% CI)^a^	*P* Value	Unadjusted OR (95% CI)		*P* Value	Adjusted OR (95% CI)^a^	*P* Value
Shoulder	0.91 (0.82-1.00)	.06	0.85 (0.77-0.95)	.003	0.4 (−8.9 to 9.8)		.92	−9.7 (−18.5 to −0.8)	.03
Neck	0.94 (0.87-1.02)	.13	0.92 (0.85-0.99)	.03	0.4 (−7.7 to 8.5)		.92	−3.8 (−10.8 to 3.3)	.30
Knee	0.87 (0.80-0.94)	<.001	0.84 (0.77-0.91)	<.001	−6.1 (−14.2 to 1.9)		.13	−10.3 (−17.8 to −2.7)	.007
Low back	0.99 (0.95-1.04)	.78	0.93 (0.88-0.98)	.004	0.6 (−5.2 to 6.5)		.83	−5.1 (−10.2 to 0.0)	.046

Abbreviation: OR, odds ratio.

^a^ Refers to analyses that were adjusted for sex, age, year of diagnosis, comorbidities (shown in Table 2), and amount of opioid use during the first 90 days after diagnosis.

Among patients who did use opioids between 91 and 365 days after the index date, our unadjusted analyses showed no difference in the case of shoulder pain (0.4%; 95% CI, –8.9% to 9.8%; *P* = .92), neck pain (0.4%; 95% CI, –7.7% to 8.5%; *P* = .92), knee pain (–6.1%; 95% CI, –14.2% to 1.9%; *P* = .13), and LBP (0.6%; 95% CI, –5.2% to 6.5%; *P* = .83) (Table 3). After adjusting for potential confounders, early physical therapy was associated with statistically significant reductions in opioid use (approximately 10%) for shoulder pain (−9.7%; 95% CI, −18.5% to −0.8%; *P* = .03), knee pain (−10.3%; 95% CI, −17.8% to −2.7%; *P* = .007), and LBP (−5.1%; 95% CI, −10.2% to 0.0%; *P* = .046). No statistically significant difference was found in the case of neck pain (−3.8%; 95% CI, −10.8% to 3.3%; *P* = .30).

The sensitivity analysis characterizing the timing associated with the benefits of early physical therapy is presented in Table 4. Receiving physical therapy between 0 and 30 days after diagnosis was associated with stronger reductions in the probability of any opioid use, except for those with knee pain. Among patients who did use opioids, receiving physical therapy between 0 and 30 days after diagnosis was associated with larger reductions (compared with the baseline analysis) in the amount of opioid use for LBP, whereas larger reductions for neck and knee pain were noted between 31 and 90 days after diagnosis.

**Table 4.** Sensitivity Analysis of the Timing of Physical Therapy and Subsequent Opioid Use^a^

Pain Site	Any Opioid Use Between 91 and 365 d After Index Date						Change in Opioid Use Between 91 and 365 d After Index Date					
	Physical Therapy Between 0 and 30 d After Index Date			Physical Therapy Between 31 and 90 d After Index Date			Physical Therapy Between 0 and 30 d After Index Date			Physical Therapy Between 31 and 90 d After Index Date		
	aOR (95% CI)	*P* Value	aOR (95% CI)		*P* Value	aOR (95% CI)		*P* Value	aOR (95% CI)		*P* Value
Shoulder	0.84 (0.72-0.97)	.02	0.87 (0.76-0.98)		.03	−6.1 (−13.8 to 1.5)		.12	3.6 (−8.7 to 16.0)		.56
Neck	0.86 (0.80-0.94)	<.001	1.14 (0.99-1.30)		.06	−4.8 (−18.1 to 8.6)		.48	−12.7 (−22.8 to −2.6)		.01
Knee	0.89 (0.79-1.00)	.04	0.80 (0.72-0.89)		<.001	2.0 (−7.7 to 11.8)		.69	−21.1 (−31.3 to −10.9)		<.001
Low back	0.89 (0.84-0.94)	<.001	1.07 (0.97-1.17)		.16	−9.6 (−15.0 to −4.0)		.001	6.1 (−3.0 to 15.3)		.19

Abbreviation: aOR, adjusted odds ratio.

^a^ All analyses shown are adjusted for sex, age, year of diagnosis, comorbidities (shown in Table 2), and amount of opioid use during the first 90 days after diagnosis.

We also performed a sensitivity analysis in which we considered an alternative definition of early physical therapy requiring 2 or more physical therapy visits within 90 days of the initial diagnosis date; the results were qualitatively similar to the baseline results (eAppendix and eTable 4 in the Supplement). Lastly, we examined the association between early physical therapy and chronic opioid use.^26^ Early physical therapy was associated with sharp reductions in chronic opioid use among patients with knee pain (OR, 0.34; 95% CI, 0.20-0.57; *P* < .001) and LBP (OR, 0.66; 95% CI, 0.57-0.76; *P* < .001), but no statistically significant association was found for shoulder pain (OR, 0.57; 95% CI, 0.32-1.04; *P* = .07) or neck pain (OR, 0.82; 95% CI, 0.63-1.06; *P* = .13).

## Discussion

Musculoskeletal pain is a common condition that imposes a substantial morbidity burden in the United States. In light of efforts to reduce opioid use in the United States, we used administrative claims data to examine whether early physical therapy was associated with decreases in long-term opioid use. Our results suggest that early physical therapy was associated with an approximate 10% reduction in the probability of any opioid use long term for patients with shoulder, neck, knee, and low back pain. For patients with low back, shoulder, and knee pain who did use opioids, early physical therapy was associated with a 5% to 10% reduction in oral MMEs. We found no association between early physical therapy and subsequent MMEs for patients with neck pain. Compared with the baseline analysis results, the sensitivity analyses findings suggest that receiving physical therapy within 0 to 30 days after the index date was associated with larger reductions in the risk of any opioid use for 3 of 4 body regions, but the data were more equivocal about whether receiving physical therapy in this study period was associated with reduction in the amount of opioid use among patients who used opioids. However, we caution that drawing definitive inferences from the sensitivity analyses is difficult because of the smaller sizes of these subgroups.

These results are largely in line with previous work for patients with LBP. In an analysis of the Military Health System Data Repository, early physical therapy was associated with reductions in opioid use,^7^ and the same pattern was noted in an analysis of claims data from New York State for individuals receiving immediate physical therapy.^10^ Our findings converged with those of previous studies, but the magnitude of the association (as measured by ORs) was smaller in this cohort. In a previous study of patients with neck pain in a single health system, early physical therapy management was associated with larger improvements in functional outcomes and pain intensity ratings.^27^ In addition, another previous study at a single institution found a decrease in the odds of opioid use 12 months later among patients with neck pain whose first clinician was a nonpharmacologic health care practitioner (ie, physical therapy or chiropractic).^28^ Similar protective effects for narcotic uses were reported in Medicare beneficiaries receiving early rehabilitation for atraumatic knee pain in ambulatory settings.^29^ We found no previous studies in shoulder pain for comparison.

This analysis expands on these previous studies by examining simultaneously a much broader set of patients (a large, national sample of privately insured patients) and the association between early physical therapy and opioid use across the 4 most common types of musculoskeletal pain conditions (neck, shoulder, low back, and knee pain). In this manner, this study may help inform the potential implications of the widespread application of the American College of Physicians and the Centers for Disease Control and Prevention guidelines for management of musculoskeletal pain. The magnitude of these findings suggest that early physical therapy may provide mild to moderate protection against the risk and intensity of long-term opioid use for patients with severe musculoskeletal pain. The sensitivity analyses provide an indication of clinical relevance or application by highlighting how receiving physical therapy within 30 days enhances protection against opioid use, and the largest protective effects were associated with limiting the number of prescriptions or supply days for knee pain or LBP. In addition, these findings suggest that early physical therapy may result in mild to moderate reductions in the intensity of opioid use by patients with shoulder and knee pain and possibly for LBP.

In contrast, early physical therapy may not reduce the intensity of opioid use for patients with neck pain. The lack of convergence between our findings and those of previous studies of neck pain could be explained by the differences in the patient populations, the resistance of many neck conditions such as whiplash to physical therapy, the underlying rate of opioid use, the timing and rate of patients receiving early physical therapy, or our decision to limit the analysis to patients with indicators of higher severity.^30,31^ Overall, our findings suggest that American College of Physicians and Centers for Disease Control and Prevention guidelines can be more confidently applied for patients with shoulder, knee, and low back pain if reducing the risk of long-term opioid use is a treatment goal.

### Limitations

These results should be viewed in light of the study limitations. First, as with all observational studies, we cannot rule out the possibility of residual confounding from unmeasured factors. However, the differences between patients who did and did not receive early physical therapy met statistical significance for most of the comorbidities we examined, and where differences existed, were small in magnitude. Second, this study captured early physical therapy, but no standard time definition exists in the literature.^5,6,7,8,9,10,11^ The definition used for this study is later than the others reported in the literature. Third, we were not able to capture specific details about the physical therapy, such as the type of approach used. Fourth, we were not able to assess the association between physical therapy and measures of function or disability, as these data are typically not provided in administrative claims databases. Fifth, this study was limited to patients who had employer-sponsored private health insurance and who were continuously enrolled for the 2-year period before and after the date of their musculoskeletal pain diagnosis.

## Conclusions

Using early physical therapy, consistent with recent clinical guidelines, could play an important role in reducing the risk of transitioning to chronic long-term opioid use for patients with shoulder, neck, knee, and low back pain. The same protective association was not identified for the intensity of opioid use in patients, particularly in patients with neck pain, suggesting that additional research is needed to determine what services mitigate long-term opioid use in neck pain. Understanding why early physical therapy appears to alleviate some types of musculoskeletal pain but not others is another high-priority area for further research. Such a study can provide insights into the implications of the widespread adoption of nonpharmacologic pain management strategies.

## Notes

Supplement. **eAppendix.** Data and Methods **eFigure.** Sample Construction Flowchart **eTable 1.** CPT Codes Used to Identify E&M and Emergency Care Visits **eTable 2.** ICD-9 and ICD-10 Codes Used to Identify Common Musculoskeletal Conditions **eTable 3.** ICD-9 and ICD-10 Codes Used to Identify Non-Musculoskeletal Reasons for Low Back Pain **eTable 4.** Additional Sensitivity Analyses
