# Supplementary material for: Association of Early Physical Therapy With Long-term Opioid Use Among Opioid-Naive Patients With Musculoskeletal Pain
Source: JAMA Netw Open. 2018 Dec 14;1(8):e185909. doi: 10.1001/jamanetworkopen.2018.5909 (PMC6324326; doi:10.1001/jamanetworkopen.2018.5909)
Supplement: Supplement. — eAppendix. Data and Methods eFigure. Sample Construction Flowchart eTable 1. CPT Codes Used to Identify E&M and Emergency Care Visits eTable 2. ICD-9 and ICD-10 Codes Used to Identify Common Musculoskeletal Conditions eTable 3. ICD-9 and ICD-10 Codes Used to Identify Non-Musculoskeletal Reasons for Low Back Pain eTable 4. Additional Sensitivity Analyses [file jamanetwopen-1-e185909-s001.pdf]

Supplementary Online Content

Sun E, Moshfegh J, Rishel CA, Cook CE, Goode AP, George SZ. Association of early physical therapy with long-term opioid use among opioid-naive patients with musculoskeletal pain. *JAMA Netw Open.* 2018;1(8):e185909. doi:10.1001/jamanetworkopen.2018.5909

eAppendix. Data and Methods

eFigure. Sample Construction Flowchart

eTable 1. CPT Codes Used to Identify E&M and Emergency Care Visits

eTable 2. ICD-9 and ICD-10 Codes Used to Identify Common Musculoskeletal Conditions

eTable 3. ICD-9 and ICD-10 Codes Used to Identify Non-Musculoskeletal Reasons for Low Back Pain

eTable 4. Additional Sensitivity Analyses

This supplementary material has been provided by the authors to give readers additional information about their work.

©2018 Sun E et al. *JAMA Network Open.*

eAppendix. Data and Methods

Data and Sample Creation. In the paper we describe how the Marketscan® data was used to construct our sample (see **eFigure 1**). In this section, we provide a more detailed description of this process. Our baseline sample consisted of continuously enrolled individuals (defined as patients with insurance coverage during the year prior to and following the index year, as well as the index year itself) with prescription drug coverage, a second outpatient visit or emergency room visit (see **eTable 1** for a list of CPT codes used) with a primary diagnosis of musculoskeletal pain (see **eTable 2**) within 30 days of the index date, as well as an opioid prescription within 90 days of the index date.

From this initial sample of 177,050 patients, we then applied the following exclusion criteria. First, we excluded individuals with an age less than 18 or greater than 64 as of the index date (n=5,131). Second, we excluded individuals having an inpatient admission with a primary diagnosis code for musculoskeletal pain or patients having an inpatient admission with any diagnosis code corresponding to an accident or trauma (all codes starting with an "E") during the entire study period, in order to exclude potential trauma patients (n=13,259). To identify a primary diagnosis, we used the PDX or DX1 fields in the inpatient admission file, and to identify trauma we used the PDX and the DX1-DX15 fields in the same file. Third, we excluded patients with a cancer diagnosis code in either the inpatient or outpatient files during the entire study period (n=5,990). A cancer condition was identified using codes DX1-DX15 in the inpatient admissions file and codes DX1-DX2 in the outpatient file. Fourth, we excluded individuals with musculoskeletal pain in more than one of the studied regions during the study period (e.g., neck and shoulder pain; n=40,523). Fifth, we excluded, individuals who had conditions that are considered confounding to low back pain during the study period (see **eTable 3** for these additional conditions, n=19,799). Similar to the identification of cancer conditions, we used DX1-DX15 in the inpatient files to identify confounding conditions, and DX1-DX2 in the outpatient files.

Sixth, we applied several exclusions related to opioid use during days 0-365 following the index date (n=2,387). Individuals were excluded if 1) we were unable to calculate MMEs for their opioid prescription, which was the case if information on drug strength was missing, 2) the total days of supply, or the total drug quantity prescribed for the period 0-90 or 91-365 was less than or equal to zero, which can occur due to clerical mistakes, or 3) the prescription was associated with a drug formulation for which the calculation of MME lacks precision. The formulations that we excluded in our sample were solutions, elixirs, and syringes. Finally, we also excluded individuals with the top one percent of MMEs during days 0-90 and during days 91-365 following the index dates, to minimize the potential effect of outliers (n=976). The 99th percentile of MMEs during days 0-90 was 4,455 and for days 91-365 the corresponding values was 18,100. Our final sample consisted of 88,985 patients.

Statistical Methods. To estimate the association between early physical therapy and subsequent opioid use, we used two sets of analysis. First, we analyzed the association between physical therapy and opioid use on the extensive margin, by estimating the likelihood that a patient will receive an opioid prescription during days 91-365 if the patient underwent early physical therapy during days 0-90. We did this using a logistic regression separately for each of the pain conditions (e.g. neck pain):

$$opioid_{i,91-365} = \delta_t + \gamma X_i + d_{i,0-90}^{0-10} + \dots + d_{i,0-90}^{90-100} + \beta_1 PT_{i,0-90} + \varepsilon_i$$

©2018 Sun E et al. *JAMA Network Open.*

where $opioid_{i,91-365}$ is and indicator of any opioid prescription for individual *i* during days 91-365 following the index date, δ_t represents year effects of year *t*, γX_i is a vector of individual characteristics including age, sex and comorbidities, $PT_{i,0-90}$ is an indicator of early physical therapy for individual *i* during days 0-90 following the index date, and ε_i is the error terms. The set of variables $d_{i,0-90}^{0-10}$ to $d_{i,0-90}^{90-100}$ are indicators of an individual's MME during days 0-90: $d_{i,0-90}^{0-10}$ takes the value 1 if any individual's MME lies in the bottom 10 percent of the overall distribution of MMEs during days 0-90, and zero otherwise. By including these dummies, we control for the intensity of opioid use during days 0-90. The regression was performed separately for each type of musculoskeletal pain (e.g. neck and shoulder pain) using robust standard errors. The main parameter of interest is β_1, which can be interpreted as the odds of a subsequent opioid prescription for patients undergoing early physical therapy. An odds ratio less than 1, indicates a reduced odd of a subsequent opioid prescription. **Table 3** in the manuscript depicts the odds ratios (β_1) separately for each of the pain conditions, as well as the associated confidence intervals.

Second, we analyzed the association between physical therapy and opioid use on the *intensive* margin; conditional on having an opioid prescription during days 91-365 we used the following multivariable linear regression to estimate the intensity of opioid use):

$$\ln(MME_{i,91-365}) = \delta_t + \gamma X_i + \alpha_1 PT_{i,0-90} + \varepsilon_i$$

where $MME_{i,91-365}$ is the oral morphine milligram equivalent during days 91-365 following the index date, δ_t and γX_i are again year effects and a vector of individual characteristics, respectively, $PT_{i,0-90}$ is an indicator of early physical therapy for individual *i* during days 0-90 following the index date, and ε_i is the error terms. The main parameter of interest is α_1, which if negative, measures the average percentage reduction in MME during days 91-365 associated with early physical therapy. **Table 3** in the manuscript depicts the percentage reduction in MME (α_1) separately for each of the pain conditions, as well as the associated confidence intervals.

©2018 Sun E et al. *JAMA Network Open.*

eFigure. Sample Construction Flowchart[a]

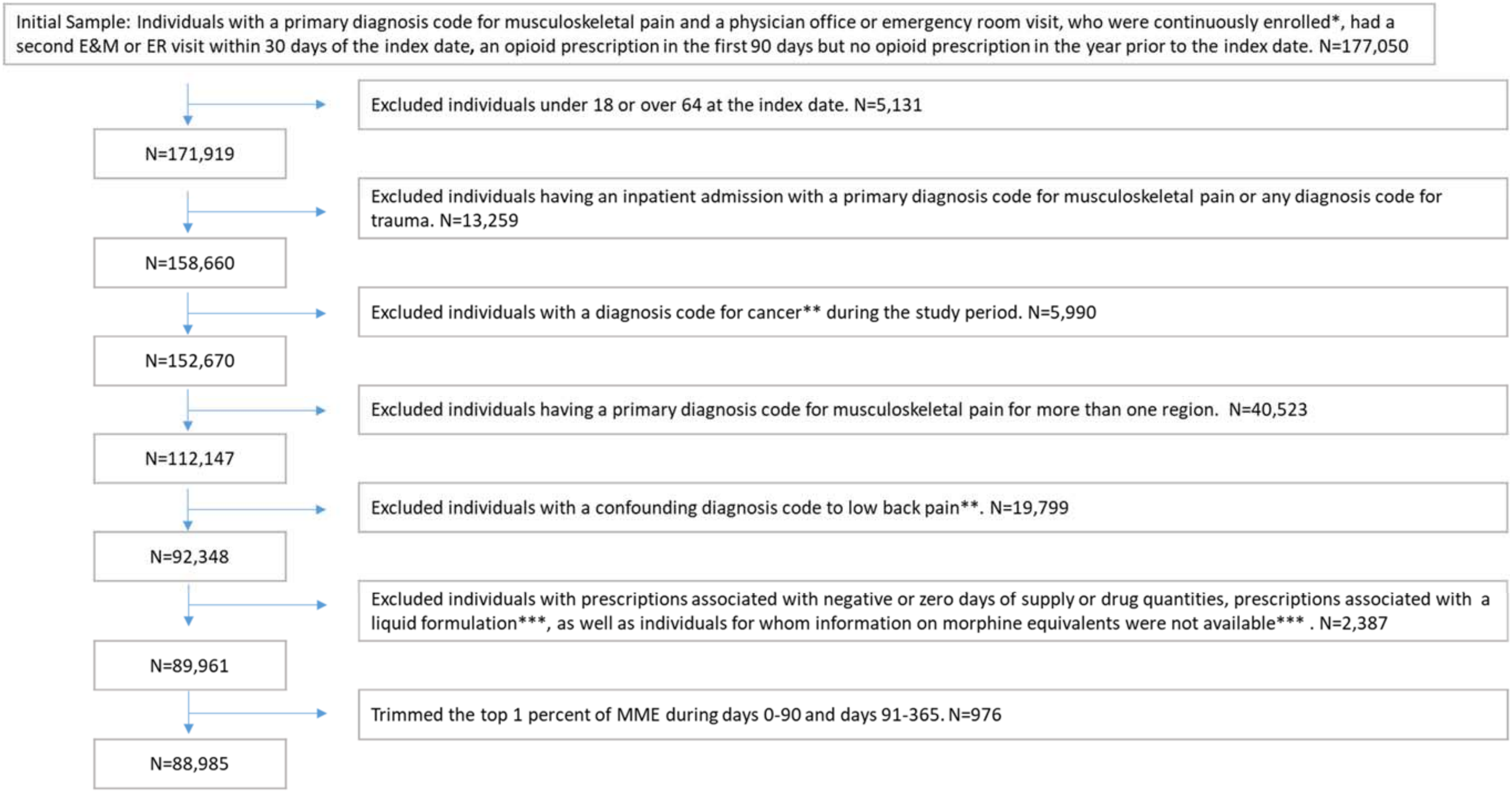

[a]This figure illustrates the exclusions that were applied to the initial sample of patients, as well as the final sample of patients used in the study. The final sample consisted of 88,985 patients.

©2018 Sun E et al. *JAMA Network Open.*

eTable 1. CPT Codes Used to Identify E&M and Emergency Care Visits[a]

Visit Type	CPT
Outpatient E&M Visit	99201, 99202, 99203, 99204, 99205, 99211, 99212, 99213, 99214, 99215, 99241, 99242, 99243, 99244, 99245, 99429, 99354, 99355, 99356, 99357, 99385, 99386, 99387, 99395, 99396, 99397, 99401, 99402, 99403, 99404, 99455, 99456, 99499, 99217, 99218, 99219, 99220, 99411, 99412, 99420, 99487, 99489, 99490
Emergency Room Visit	99281,99282, 99283, 99284, 99285

[a]Patients were included in our sample if they had an outpatient (Evaluation and Management; E&M) visit or emergency room visit with a diagnosis code for musculoskeletal neck, knee, shoulder, or low back pain. This table shows the procedure (CPT) codes used to identify outpatient and emergency room visits.

©2018 Sun E et al. *JAMA Network Open.*

eTable 2. ICD-9 and ICD-10 Codes Used to Identify Common Musculoskeletal Conditions[a]

Neck		*Shoulder*		*Knee*		*Low Back Pain*	
**ICD-9**	**ICD-10**	**ICD-9**	**ICD-10**	**ICD-9**	**ICD-10**	**ICD-9**	**ICD-10**
721.0	M47.812	726.1	M75.100, M75.50	715.16	M17.10	721.3	M47.817
721.1	M47.12	727.61	M75.120	715.26	M17.5	722.10	M51.26, M51.27
722.0	M50.20	840.4	S43.429A	715.36	M17.9	722.52	M51.36, M51.37
722.4	M50.30	726.2	M75.30, M75.40, M75.80	715.96	M17.9	722.73	M51.06, M51.07
722.71	M50.00	726.10	M75.100, M75.50	716.46	M12.869	722.93	M46.47, M51.86, M51.87
722.81	M96.1	726.11	M75.30	716.56	M13.0	724.02	M48.06
722.91	M50.80, M50.90			716.66	M13.169	724.2	M54.5
723.0	M48.02			716.96	M12.9	724.3	M54.30
723.1	M54.2			717.7	M22.40	724.4	M54.14 – M54.17
723.2	M53.0			719.46	M25.569	724.5	M54.89, M54.9
723.3	M53.1			719.56	M25.669	756.11	Q76.2
723.4	M54.12, M54.13			719.96	M25.9	756.12	Q76.2
723.5	M43.6					846.0	S33.8XXA
723.6	M54.02					846.1	S33.6XXA
723.7	M67.88					846.8	S33.8XXA
723.8	M53.82					846.9	S33.9XXA
723.9	M53.82					847.2	S33.5XXA
739.0	M99.00					847.3	S33.8XXA
739.1	M99.01						
847.0	S13.4XXA, S13.8XXA						

[a]This table shows the diagnosis codes used to identify patients with musculoskeletal pain conditions in the specified body regions. Conversions from ICD-9 to ICD-10 were made using the following source: http://www.icd10codesearch.com/

©2018 Sun E et al. *JAMA Network Open.*

eTable 3. ICD-9 and ICD-10 Codes Used to Identify Non-Musculoskeletal Reasons for Low Back Pain[a]

ICD-9 code	ICD-10	Description
592.xx	N20.0, N20.1, N20.9	Calculus of kidney
574.20	K80.20	Calculus of gallbladder without mention of cholecystitis
599.0	N39.0	Urinary tract infection, site not specified
V13.02	Z87.440	Urinary (tract) infection
140.xx – 239.xx	C00.xx – D49.xx	Neoplasms
V17.81, V82.81	Z82.62, Z13.820	Osteoporosis
344.6	G83.4	Cauda equine syndrome
730.xx	M86.xx, M89.60, M89.619, M89.629, M89.639, M89.649, M89.659, M89.669, M89.679, M89.968, M89.69, M90.80, M90.810, M90.829, M90.830, M90.849, M90.859, M90.869, M90.879, M90.88, M90.89, M46.20, M46.30	Osteomyelitis, periostitis, and other infections involving bone
731.3	M89.70	Major osseous deficit

[a]Patients with low back pain were excluded from the sample if they also had one of the diagnosis codes listed above, as these conditions can be confused with musculoskeletal pain. Conversions from ICD-9 to ICD-10 were made using the following source: http://www.icd10codesearch.com/

©2018 Sun E et al. *JAMA Network Open.*

eTable 4. Additional Sensitivity Analyses[a]

Pain Site	Adjusted Odds Ratio (95%CI)	Adjusted % Change in Opioid Use (95% CI)
Early physical therapy defined as 2 or more physical therapy visits within 90 days of initial diagnosis		
Shoulder	0.88<br>[0.79 to 0.98]<br>p=0.023	-12.3<br>[-21.3 to -3.2]<br>p=0.008
Neck	0.90<br>[0.83 to 0.97]<br>p=0.010	-2.3<br>[-9.7 to 5.0]<br>p=0.54
Knee	0.81<br>[0.74 to 0.88]<br>p<0.001	-9.6<br>[-17.6 to -1.8]<br>p=0.016
Low Back	0.94<br>[0.89 to 0.99]<br>p-=0.021	-4.8<br>[-10.2 to 0.5]<br>p=0.08
Outcome is Chronic Opioid Use (≥10 prescriptions or ≥120 days supply during days 91-365 after the initial diagnosis		
Shoulder	0.57<br>(0.32 to 1.04)<br>p=0.07	N/A
Neck	0.82<br>(0.63 to 1.06)<br>p=0.13	N/A
Knee	0.34<br>(0.20 to 0.57)<br>p<0.001	N/A
Low Back	0.66<br>(0.57 to 0.76)<br>op<0.001	N/A

[a]This table presents the results of two sensitivity analyses. The first defined early physical therapy as having received 2 or more physical therapy visits during the first 90 days after initial diagnosis. For this analysis, we present the adjusted odds ratio for filling at least 1 opioid prescription during days 91-365 following the initial diagnosis as well as the adjusted % decrease in opioid use (measured in oral morphine equivalents) among patients who used opioids. The second sensitivity analysis used an alternative outcome: chronic opioid use, defined as having filled (≥10 prescriptions or ≥120 days supply during days 91-365 after the initial diagnosis. For this analysis, we present the adjusted odds ratio for chronic opioid use. "Adjusted" refers to analyses that adjusted for sex, age, year of diagnosis, the comorbidities shown in Table 2, and the amount of opioid use in the first 90 days after diagnosis.

©2018 Sun E et al. *JAMA Network Open.*
